# Retention in care of people on antiretroviral therapy who inject drugs in South Africa

**DOI:** 10.4102/sajhivmed.v26i1.1710

**Published:** 2025-10-29

**Authors:** Phumzile C. Mngomezulu, Rifqah A. Roomaney, Brian E. van Wyk

**Affiliations:** 1 School of Public Health, Faculty of Community and Health Sciences, University of the Western Cape, Cape Town, South Africa; 2 Burden of Disease Research Unit, South African Medical Research Council, Cape Town, South Africa

**Keywords:** HIV, AIDS, housing, opioid substitution therapy, people who inject drugs, retention in care

## Abstract

**Background:**

Retention of people who inject drugs (PWID) on antiretroviral therapy (ART) is critical for viral suppression. However, PWID, a key population, traditionally have poor retention in care (RiC).

**Objectives:**

To determine the prevalence of and factors associated with RiC at 6 months, following ART initiation in three South African districts.

**Method:**

Data of 333 PWID (adults 18+ years), who commenced ART between July 2022 and March 2023, were retrieved from TIER.Net electronic database.

**Results:**

RiC after 6 months on ART was 40% (*n* = 132). Bivariate analysis showed higher retention among those on Opioid Substitution Therapy (OST) with treatment support compared to those without support (95% vs 39%; *P* < 0.001); and lower RiC among those with unstable housing compared to those with stable housing (12% vs 75%; *P* < 0.001). In the survival analysis, PWID with unstable housing had a 5-fold increased risk of poor RiC (adjusted hazard ratio [AHR] = 4.94; 95% confidence interval [95% CI]: 2.35–10.35), while those in OST had a 75% decreased risk of poor RiC (AHR = 0.25; 95% CI: 0.10–0.60).

**Conclusion:**

PWID face significant challenges in remaining engaged in ART care, particularly those experiencing unstable housing. OST uptake can facilitate improved RiC and health outcomes, highlighting the need for expanded harm reduction strategies. Addressing unstable housing remains urgent to strengthen HIV treatment outcomes for PWID in South Africa.

**What this study adds:** This study highlights the critical role of Opioid Substitution Therapy and stable housing in improving retention of PWID on ART in South Africa. It provides evidence for integrating harm reduction and housing support into HIV care models to enhance treatment outcomes for this key population.

## Introduction

Recreational drug injection has been reported in approximately 158 countries, with 123 of these countries also reporting HIV infections among people who inject drugs (PWID).^1^ Because of the practice of sharing injecting equipment, PWID are at a significantly higher risk of contracting HIV.^1^ Despite this elevated risk, these populations often remain marginalised and have limited access to health and social services.^2^ Globally, it is estimated that 1.6 million out of the 12 million PWID (13%) are living with HIV.^2^ In 2015, over 150 000 PWID contracted HIV, and 60 000 died from AIDS-related complications in the same year.^3^

A study in three large South African cities (Cape Town, Johannesburg, and Pretoria) found that 14% of people living with HIV were PWID.^4^ Despite the notable growth in the illicit drug trade to South Africa and the growing prevalence of HIV, no studies have been conducted on HIV among PWID and related risk factors since 2016.^4^ Over the past decade, few studies have investigated HIV and associated risk behaviour among PWID in South Africa.^5,6,7^ Furthermore, only two of these studies recruited more than 50 PWID.

Harm reduction is an evidence-based method that focuses on involving people who use and inject drugs and providing them with life-saving equipment and information to create behaviour change.^8^ There is substantial evidence that harm-reduction initiatives save money and are cost-effective, improving mental, physical and social well-being of PWID.^8^ Harm reduction is a fundamental tool for organising the fight against the opioid crisis, HIV/AIDS, viral hepatitis, and other illnesses.^8^ South Africa has only four harm reduction projects aimed at managing HIV in people who use and inject drugs, which function separately as non-governmental organisations with limited to no integration with public primary healthcare facilities.^9^

### Objectives

This study reports on the retention in care (RiC) of PWID, 18+ years, who commenced antiretroviral therapy (ART) in Ehlanzeni, Tshwane and eThekwini districts, South Africa, between July 2022 and March 2023, and the factors associated with RiC after 6 months post-ART initiation.

## Research methods and design

### Design

We conducted a retrospective cohort analysis of PWID, 18+ years, who commenced ART between July 2022 and March 2023 in Ehlanzeni, Tshwane and eThekwini districts.

### Study context

The study was conducted at three TB HIV Care Organisation PWID key population sites, namely Tshwane, eThekwini and Ehlanzeni municipal districts. Tshwane District is situated in Pretoria, Gauteng province. In 2017, Tshwane district had an estimated population of 3 555 741 across all seven Tshwane sub-districts with an HIV prevalence of 10.5% and 141 436 people enrolled in ART programmes.^10^ The approximate population of PWID in Tshwane is 3896, which was estimated through mapping done by the TB HIV Care Organisation PWID Programme in 2019. In Tshwane, TB HIV Care is the only referral site for HIV management and harm-reduction services designed specifically for PWID as a key population. eThekwini is situated in KwaZulu-Natal province. It has the country’s largest port, with a population of 3 702 231 estimated in 2016.^11^ In 2019, an estimate of 1245 PWID was used to inform the programme in the district. The district accounts for 17% of the KwaZulu-Natal province.^12^ Ehlanzeni is situated in Mpumalanga province, with four municipalities, namely the city of Mbombela, Bushbuckridge, Nkomazi and Thaba Chweu. The estimated population was 1 917 082 with 1087 estimated PWID population in 2019.^13^ The HIV prevalence in the Mpumalanga province was 17.3% with 20% in Ehlanzeni District.^14^

The programme focuses on HIV prevention and management among PWID through the implementation of harm-reduction strategies. The programme employs recovering drug injectors as peer educators who work alongside health professionals to promote relatability, advocacy and targeted service provision to the PWID population.

The three PWID sites provide a comprehensive harm reduction service package that is tailor-made to address the needs of PWID. This key population is reached through the drop-in centre (DIC) and daily mobile outreach services. The sites are well equipped with trained doctors, Nurse-Initiated Management of Anti-retroviral Therapy (NIMART) nurses, social workers, linkage tracers and peer educators that are trained and certified to conduct HIV counselling and testing.

### Data source

This analysis utilised two data sources: Three Interlinked Electronic Registers (TIER.Net) and an Opioid Substitution Therapy (OST) Microsoft Excel spreadsheet. TIER.Net is a software used to store routine facility-level HIV patient data. This database is linked to the District Health Information System (DHIS) for district-level periodic reporting. All three settings indicated in the study have adopted TIER.Net for easy monitoring of large cohorts. The OST Microsoft Excel spreadsheet was reviewed to determine the OST status of all the clients on ART during the study period. The OST spreadsheet consisted of treatment support information captured for every participant that was enrolled in OST. Data on treatment support were only measured in participants who were currently enrolled in OST.

### Study participants

Data for 333 PWID initiated on ART were found. An all-inclusive census sampling was employed.

### Main outcome measures and analysis

Data on sociodemographic variables were obtained (age, gender and housing), clinical characteristics (Baseline CD4 count, duration on ART, and enrolment on OST), and psychosocial characteristics (Treatment supporter). In accordance with the OST psychosocial support guidelines, a treatment supporter such as a next of kin is defined as an individual who assists in facilitating the treatment process of the service user. Treatment supporters play a critical role in OST management, as evidence indicates they enhance treatment adherence.

A PWID was considered non-RiC if they missed 3 consecutive months of follow-up since their last visit to an HIV treatment and care facility. Although monthly follow-up is not routine practice in ART clinics, the PWID programme monthly follow-up visits allowed for a consistent tracking of service engagement and enhanced support. RiC was therefore defined as adherence to monthly follow-up visits for at least 6 months post-ART initiation.

Descriptive and inferential statistics were used to summarise and describe these characteristics using Statistical Package for Social Sciences (SPSS) version 28 (IBM Corp., Armonk, New York, United States) and Stata 18 (StataCorp, College Station, Texas, United States). We employed bivariate analysis to determine the significance and strength of association between RiC and various sociodemographic, clinical and psychosocial characteristics at *P* < 0.05. Survival analysis was assessed with non-RiC as the outcome of interest. We constructed crude survivor curve graphs to visually depict the survivor function (non-RiC) of housing and enrolment on OST variables. We employed log-rank tests to test associations with RiC. After this initial exploration, a further investigation aimed at identifying risk/protective factors and determinants associated with RiC and non-RiC at 6 months was undertaken utilising Cox proportional hazard models, both in their crude and adjusted forms. Adjusted hazard ratios (AHR) and their corresponding 95% confidence intervals (95% CI) were calculated to quantify the strength of these associations.

### Ethical considerations

To maintain anonymity and confidentiality personal information was de-identified. Additionally, data were password-protected, stored electronically, and reported in aggregate form. Approval for degree purposes was obtained from the University of the Western Cape’s Senate Higher Degrees Committee and ethical clearance was granted by the University of the Western Cape Biomedical Research Ethics Committee (refence number: BM23/8/5).

## Results

Of the 333 PWID and who were newly initiated on ART between July 2022 and March 2023, the median age was 33.5 years (interquartile range: 29–37) and the majority were men (*n* = 310; 93%) (Table 1). Most participants were living in unstable housing (*n* = 187; 56%). Some of the records on the baseline CD4 variable were missing. Of 333 participants, only 194 had captured baseline CD4 count results. Most of the participants (*n* = 98; 51%) had an initial CD4 count recorded between 200 cells/mm^3^ and 500 cells/mm^3^ which indicated mild to moderate immunosuppression. A third of the participants (*n* = 109; 33%) were enrolled on OST. As per the OST psychosocial support policy, the majority (*n* = 76; 70%) had registered treatment supporters.

**TABLE 1:** Retention in care at 6 months by sociodemographic and clinical characteristics of people who inject drugs on antiretroviral therapy in three districts in South Africa (*N* = 333).

Description/category	Total				Retained in care (*n* = 132)				Not retained (*n* = 201)				*P*
	*n*	%	Median	IQR	*n*	%	Median	IQR	*n*	%	Median	IQR	
**Age (years)**	-	-	33.5	29–37	-	-	33	29–37	-	-	33	29–37	0.651
**Gender**	-	-	-	-	-	-	-	-	-	-	-	-	0.922
Male	310	93.1	-	-	123	93	-	-	187	93	-	-	-
Female	23	6.9	-	-	9	7	-	-	14	7	-	-	-
**Housing**	-	-	-	-	-	-	-	-	-	-	-	-	< 0.001*
Stable	146	43.8	-	-	110	83	-	-	36	18	-	-	-
Unstable	187	56.2	-	-	22	17	-	-	165	82	-	-	-
**Baseline CD4 count (cells/mm^3^)**	194	-	-	-	-	-	-	-	-	-	-	-	0.0002*
< 200	30	15.0	-	-	8	6	-	-	22	11	-	-	-
200–500	98	51.0	-	-	62	47	-	-	36	18	-	-	-
> 500	66	34.0	-	-	46	35	-	-	20	10	-	-	-
**Currently enrolled in OST**	-	-	-	-	-	-	-	-	-	-	-	-	< 0.001*
Yes	109	67.3	-	-	84	64	-	-	25	12	-	-	-
No	224	32.7	-	-	48	36	-	-	176	88	-	-	-
**Treatment supporter**	109	-	-	-	-	-	-	-	-	-	-	-	< 0.001*
Yes	76	30.3	-	-	71	54	-	-	5	2	-	-	-
No	33	69.7	-	-	13	10	-	-	20	10	-	-	-

Note: *P*-values are calculated for retained in care versus not retained in care. Chi-square test used for categorical variables and Kruskal-Wallis test used for numerical variables (e.g. age).

ART, antiretroviral therapy; IQR, interquartile range; OST, Opioid Substitution Therapy.

* , denotes statistical significance at *P* < 0.05.

Throughout the study, RiC did not improve, with 40% (*n* = 132) of PWID retained in care after 6 months post-ART initiation. Table 1 illustrates the percentage of participants retained and those who were not retained in care in each category. Gender and age were not predictors of RiC (*P* = 0.651). Significantly higher number of PWID with stable housing (*n* = 110; 83%) were retained in care, compared with those living in unstable housing (*n* = 22; 17%) (*P* ≤ 0.001).

Table 2 shows that the probability of non-RiC increased among those with unstable housing compared to those with stable housing (AHR = 4.94; 95% CI: 2.35–10.35). However, probability of non-RiC was significantly lower among those enrolled on OST compared to those not on OST (AHR = 0.25; 95% CI: 0.10–0.60).

**TABLE 2:** Predictors of non-retention in care of people who inject drugs on antiretroviral therapy in three district municipalities in South Africa (*N* = 333).

Predictor	Category	Crude		Adjusted	
		Hazard ratio	95% CI	Hazard ratio	95% CI
Housing	Stable	1.00	-	1.00	-
	Unstable	6.81	4.23–11.02	4.94	2.36–10.37
OST	No	1.00	-	1.00	-
	Yes	0.15	0.08–0.26	0.26	0.11–0.61

OST, Opioid Substitution Therapy; CI, confidence interval.

PWID that had treatment support were more likely (*n* = 71; 54%) to be retained in care compared with those without treatment support (*n* = 13; 10%).

**FIGURE 1:**
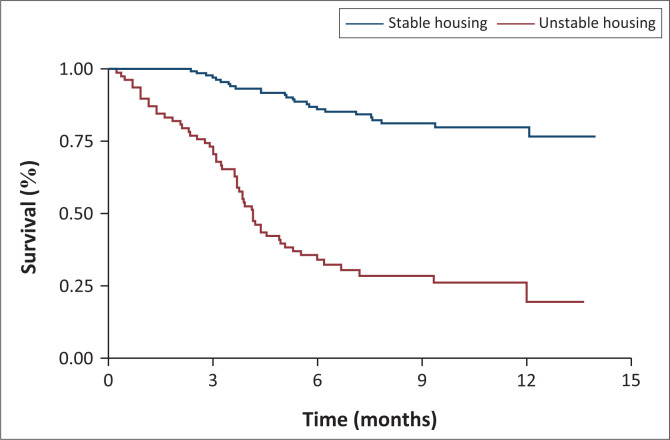
Retention in care by housing at 3 months, 6 months and 12 months.

**FIGURE 2:**
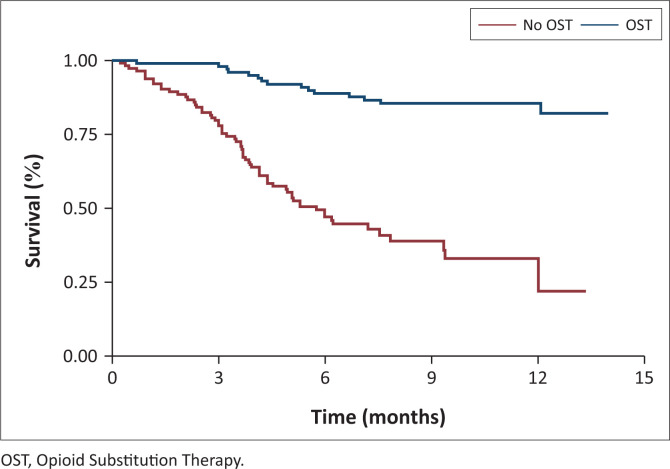
Retention in care by Opioid Substitution Therapy at 3 months, 6 months and 12 months.

**FIGURE 3:**
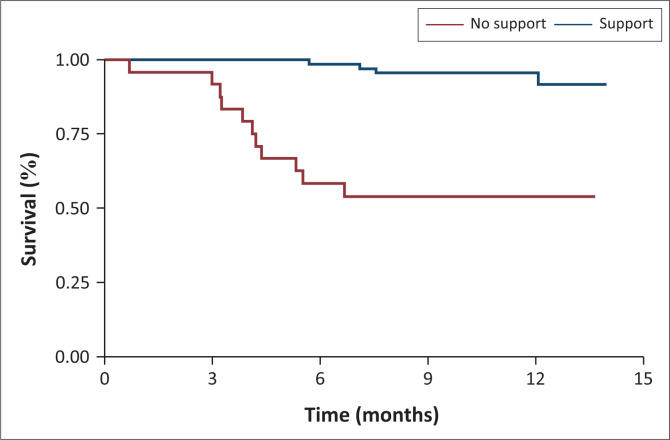
Retention in care by support at 3 months, 6 months and 12 months.

RiC over time varied significantly by housing status, enrolment on OST, and availability of treatment supporters, as illustrated in Figure 1, Figure 2, and Figure 3. At month 3, all participants in stable housing were retained in care versus 75% in unstable housing. At month 6 retention was 85% (stable) versus 27% (unstable) and at month 12, 80% (stable) versus 22% (unstable) (Figure 1). At month 3, retention was 98% for participants on OST versus 80% for those not on OST. By month 6, retention was 90% (OST) versus 50% (non-OST), and at month 12, 85% (OST) versus < 25% (non-OST) (Figure 2). At month 3, retention was 100% among participants with treatment supporters versus 90% without. At month 6, retention was 98% (with support) versus 60% (without support, and at month 12, 90% (with support) versus 60% (without support) (Figure 3).

## Discussion

In 2014, UNAIDS developed the fast-track approach that drives the 90-90-90 targets.^15^ The targets increased to 95-95-95 in 2024, with the goal of ending the HIV epidemic as a public health threat by 2030. This includes the goal that 95% of people living with HIV and on ART should be retained in care. While PWID are successfully tested and initiated on ART, retaining at least 95% of them in care is an ongoing challenge. Our study reports an alarmingly lower (40%) RiC rate throughout the observation period. Although a study by Ryan et al. in Madrid, Spain, reported a higher retention rate of 80% among people who use drugs (PWUD) and PWID, this figure declined to 62.9% by the end of 2020.^16^ Similarly, a cross-sectional study conducted in the United States reported a 77% retention rate among PWID.^17^ While these rates appear higher numerically compared to our findings, it is important to note that none of the studies met the UNAIDS 90-90-90 (now 95-95-95) target, which calls for 90% of individuals on treatment to be retained in care and achieve viral suppression. PWID traditionally experience poorer clinical outcomes and higher mortality rates. Mutai et al.’s global meta-analysis on suboptimal retention in drug users reported a 65.6% retention rate among PWID on ART, with lower viral suppression (61%) compared to the general population.^18^

The findings for gender and RiC must be interpreted with caution because of the low number of female participants enrolled in this cohort study. In this study, Cox proportional hazards analysis was used to assess RiC by gender. Out of a total of 333 participants, 132 (40%) were RiC while 201 (60%) were not retained. To explore gender-specific retention, we calculated the proportion of male and female participants among those retained and not retained in care. Of the 132 participants RiC, 123 (93%) were men and *n* = 9 (7%) were women. Similarly, among the 201 participants not RiC, 187 (93%) were men and 14 (7%) were women. The Cox model indicated no statistically significant difference (*P* = 0.922) in retention between male and female participants. Although gender was not associated with RiC, a study conducted in Vancouver, Canada, demonstrated that female PWID are approximately 30% less likely to be retained on ART because of being more vulnerable and engaged in survival activities, such as the sex trade.^19^ Furthermore, the study was considered the first long-term study to assess key demographic and behavioural factors associated with ART among PWID.^19^

In our current study, stable housing was a significant determinant of RiC among PWID. Most of the participants (*n* = 187; 56%) were homeless or living in unstable housing, with 44% (*n* = 146) living in stable housing. These findings are consistent with those from the National HIV Behavioural Surveillance (NHBS) which found that 68% of PWID in the three United States cities were homeless, with only 32% living in stable housing.^9^ Among those who were homeless, 64% of them were living with HIV while 36% of the homeless PWID were HIV negative.^9^ Our finding, which indicates that more PWID are living in unstable housing (*n* = 187; 56%) compared to those in stable housing (*n* = 146; 44%), is supported by a systematic review from 16 countries that was conducted on homelessness and the risk of HIV and poor RiC among PWID which revealed that PWID are more likely to be homeless.^20^ Furthermore, the systematic review revealed poor outcomes related to ART adherence and viral suppression.

Our findings indicate that PWID with mild to moderate immunosuppression (CD4 count of 200 cells/mm^3^ – 500 cells/mm^3^) had better chances of being retained compared to those with severe immunodeficiency (CD4 count < 200 cells/mm^3^). These results are consistent with a study conducted in KwaZulu-Natal, South Africa, which reported that 30% of participants with CD4 counts below 200 cells/mm^3^ were lost to follow-up, compared to 24.8% among those with CD4 counts between 250 cells/mm^3^ and 500 cells/mm^3^.^21^ Additionally, only 16% of participants with CD4 counts greater than 500 cells/mm^3^ were lost to follow-up.^21^ These findings indicate that higher CD4 counts are positively associated with RiC, while lower CD4 counts are predictors of loss to follow-up. CD4 count is an important indicator used to monitor the depletion of CD4 cells, efficacy of ART and disease progression. In this study, data on baseline CD4 count was obtained for only 58% (*n* = 194) of the participants. Difficulties in obtaining blood samples from PWID have been identified in some studies. Moreover, venous degradation was identified as a common complication of prolonged injection and high heroin formulation for PWID.^22^ This can lead to thrombophlebitis which makes vascular access difficult in clinical settings. As a result, missing data on a CD4 cell count may be expected in health settings that service PWID.

The current study showed that enrolment in OST was strongly associated with RiC. This study’s results align with a meta-analysis which suggests that OST increased the uptake of ART, RiC, and viral suppression among PWID.^23^ Furthermore, the systematic review advocated the concurrent scale-up of OST and ART to enhance RiC. Over the last four decades, Western countries and those in Southeast Asia have made strides in research, resource development and governing political support to broaden treatment approaches by integrating OST into national strategies amidst the opioid crisis. However, in South Africa, although the National Department of Health (NDoH) has outlined an OST plan for future provision, nationwide implementation has yet to be achieved. Consequently, only a select few have access to OST through personal finance or community-driven efforts, leaving many without this vital treatment which has proved to enhance retention in HIV care among PWID.

This study confirmed the association between treatment support and RiC. Results revealed that participants with treatment supporters were retained in care. Studies have shown that people living with HIV require robust family and societal support.^24,25^ Additional research indicates that the assistance offered by family members yields various beneficial effects on both people living with HIV and family dynamics, fostering positive health outcomes. Other studies revealed that people on OST, owing to opioid dependence, require social network support, namely family, a partner, and peers.^25^ These social networks are important in fostering treatment adherence and retention thus improving health outcomes of PWID.^25^

## Conclusion

Our study highlights low RiC among PWID at 6 months post-ART initiation. Notably, PWID are often left behind in effectively accessing HIV services. The combination of high-quality prevention programmes for PWID, including needle and syringe programme, psychosocial support, OST and ART initiation and retention should be implemented on a public health scale to potentially manage the ongoing epidemic.
